# Reappraisal of Reported Genes for Sudden Arrhythmic Death: Evidence-Based Evaluation of Gene Validity for Brugada Syndrome

**DOI:** 10.1161/CIRCULATIONAHA.118.035070

**Published:** 2018-09-17

**Authors:** S. Mohsen Hosseini, Raymond Kim, Sharmila Udupa, Gregory Costain, Rebekah Jobling, Eriskay Liston, Seema M. Jamal, Marta Szybowska, Chantal F. Morel, Sarah Bowdin, John Garcia, Melanie Care, Amy C. Sturm, Valeria Novelli, Michael J. Ackerman, James S. Ware, Ray E. Hershberger, Arthur A.M. Wilde, Michael H. Gollob

**Affiliations:** 1 Ted Rogers Cardiac Genome Clinic (S.M.H., R.K., R.J., E.L., S.B.), The Hospital for Sick Children, Toronto, Ontario, Canada.; 2 Division of Clinical and Metabolic Genetics (R.K., G.C., R.J., E.L., S.M.J., S.B.), The Hospital for Sick Children, Toronto, Ontario, Canada.; 3 Toronto General Hospital Research Institute, University of Toronto, Ontario, Canada (S.U., M.H.G.).; 4 Fred A. Litwin Family Center in Genetic Medicine, University Health Network, Toronto, Ontario, Canada (R.K., M.S., C.F.M.).; 5 Invitae Corporation, San Francisco, CA (J.G.).; 6 Peter Munk Cardiac Centre, Department of Medicine (M.C., M.H.G.), Toronto General Hospital, University of Toronto, Ontario, Canada.; 7 Department of Physiology, Peter Munk Cardiovascular Molecular Medicine Laboratory (M.H.G.), Toronto General Hospital, University of Toronto, Ontario, Canada.; 8 Geisinger Health System Genomic Medicine Institute, Danville, PA (A.C.S.).; 9 Centro Benito Stirpe per la Morte Improvvisa del Giovane Atleta, Fondazione Policlinico Universitario Agostino Gemelli, Catholic University of the Sacred Heart, Rome, Italy (V.N.).; 10 Departments of Cardiovascular Diseases, Pediatrics, and Molecular Pharmacology and Experimental Therapeutics, Divisions of Heart Rhythm Services and Pediatric Cardiology, Windland Smith Rice Sudden Death Genomics Laboratory, Rochester, MN (M.J.A.).; 11 National Heart and Lung Institute, MRC London Institute of Medical Sciences, Imperial College London, Royal Brompton & Harefield Hospitals, United Kingdom (J.S.W.).; 12 Department of Internal Medicine, Division of Human Genetics and Cardiovascular Division, Ohio State University, Columbus (R.E.H.).; 13 AMC Heart Center, Department of Clinical and Experimental Cardiology, Academic Medical Center, Amsterdam, The Netherlands (A.A.M.W.).; 14 Princess Al-Jawhara Al-Brahim Centre of Excellence in Research of Hereditary Disorders, Jeddah, Saudi Arabia (A.A.M.W.). Columbia University Irving Medical Centre, New York (A.A.M.W.).; 15 * Drs Hosseini, Kim, and Udupa contributed equally.

**Keywords:** Brugada syndrome, genetics, sudden death

## Abstract

**Background:**

Implicit in the genetic evaluation of patients with suspected genetic diseases is the assumption that the genes evaluated are causative for the disease based on robust scientific and statistical evidence. However, in the past 20 years, considerable variability has existed in the study design and quality of evidence supporting reported gene-disease associations, raising concerns of the validity of many published disease-causing genes. Brugada syndrome (BrS) is an arrhythmia syndrome with a risk of sudden death. More than 20 genes have been reported to cause BrS and are assessed routinely on genetic testing panels in the absence of a systematic, evidence-based evaluation of the evidence supporting the causality of these genes.

**Methods:**

We evaluated the clinical validity of genes tested by diagnostic laboratories for BrS by assembling 3 gene curation teams. Using an evidence-based semiquantitative scoring system of genetic and experimental evidence for gene-disease associations, curation teams independently classified genes as demonstrating limited, moderate, strong, or definitive evidence for disease causation in BrS. The classification of curator teams was reviewed by a clinical domain expert panel that could modify the classifications based on their independent review and consensus.

**Results:**

Of 21 genes curated for clinical validity, biocurators classified only 1 gene (*SCN5A*) as definitive evidence, whereas all other genes were classified as limited evidence. After comprehensive review by the clinical domain Expert panel, all 20 genes classified as limited evidence were reclassified as disputed with regard to any assertions of disease causality for BrS.

**Conclusions:**

Our results contest the clinical validity of all but 1 gene clinically tested and reported to be associated with BrS. These findings warrant a systematic, evidence-based evaluation for reported gene-disease associations before use in patient care.

## Abstract 2

Supplemental Digital Content is available in the text.

**Clinical Perspective**

**What Is New?**

- This evidence-based review of genes reported to cause Brugada Syndrome (BrS) and routinely clinically tested in patients indicates that 20 of 21 genes lack sufficient genetic evidence to support their causality for BrS.
- Only the *SCN5A* gene is classified as having definitive evidence as a cause for BrS.

**What Are the Clinical Implications?**

- Routine genetic evaluation of genes other than *SCN5A* is not currently warranted in the clinical care of patients with BrS.
- Genetic testing of genes without sufficient evidence supporting causality for BrS may lead to incorrect interpretation of rare variants in these genes and inappropriate diagnostic conclusions or interventions for patients and family members.

**Editorial, see p 1206**

The Human Genome Project imbued science and medicine with the blueprint for human health and disease.^1^ In the last 25 years, the evolution of this historic accomplishment has been extraordinary, enabling the elucidation of the genetic and molecular underpinning of thousands of human diseases. The impact on healthcare delivery has been extensive and has defined a new era of precision medicine. Now patients may have their clinical diagnosis genetically confirmed, in some cases specifically managed based on genotype, and may choose to share their genetic information with at-risk family members to allow for presymptomatic genetic testing for disease risk. Provision of genetic testing services for clinical care is now widely available, and currently 54 057 genetic tests offered by 503 labs are recorded by the Genetic Testing Registry of the National Center for Biotechnology Information (https://www.ncbi.nlm.nih.gov/gtr/ queried October 12, 2017), examining 16 236 genes for 10 889 conditions.

Successful implementation of precision medicine, at its foundation, necessitates that reported gene-disease associations are reliably evidence-based to ensure the appropriate application of genetic information in optimizing care while preventing inaccurate conclusions that may cause harm. There remains considerable variability in the level of genetic and experimental evidence of reported gene-disease associations, raising questions about the clinical validity of some genes and potential concerns at their inclusion for clinical genetic testing. Available gene-disease databases such as Online Mendelian Inheritance in Man,^2^ although valuable, lack the rigorous critical approach to examine the clinical validity of proposed associations. The need for a systematic, evidence-based method for curating gene-disease associations spurred the development of ClinGen (Clinical Genome Resource),^3^ a National Institute of Health-funded international consortium of geneticists, genomic scientists, and clinical domain experts, with the common goal of defining standardized, evidence-based frameworks for assessment of reported genetic associations for use in precision medicine.

Here, we report the first application of the ClinGen evidence-based gene curation framework for sudden cardiac death-predisposing, genetic heart rhythm diseases. Brugada syndrome (BrS) has an estimated prevalence of 1:2000.^4,5^ When familial, it follows an autosomal-dominant mode of inheritance.^6^ To date, >20 genes have been reported to be associated with BrS^6^ and are routinely tested as single-gene causes for this condition on a variety of clinical genetic testing panels worldwide. Because disease penetrance for BrS is incomplete and age-related, genetic testing may be used for diagnostic purposes and for the screening of at-risk family members. In this context, genetic information may lead to disease labeling in individuals, influence physicians’ decision making in guiding preventative treatments by means of an implantable cardioverter defibrillator, or lead to cascade screening of family members. In view of the significant impact a diagnosis of BrS (or any genetic disease) may have on an individual and his or her family, it is critical that only genes with robust clinical validity be evaluated in the care of patients to minimize the risk of incorrect interpretation of genetic information that may ultimately cause undue harm or anxiety.

## Methods

For purposes of replicating the results or process of this study, the analytic methods and study materials for this study are described and referenced accordingly, and all data are available to other researchers in the online-only Data Supplement(scores).

### Selection of Genes for Curation

Genes were selected for curation if they met all of the following criteria: (1) ≥2 publications in peer-reviewed medical literature suggesting single-gene causality for BrS, (2) reported literature presenting both genetic and experimental data, and (3) present on ≥3 BrS clinical genetic testing panels from accredited diagnostic laboratories. It should be noted that a number of the genes evaluated for BrS have also been implicated in other diseases; however, this effort did not evaluate the validity of any gene for disorders other than BrS.

### Gene Curation Framework

We formed 3 gene curation teams to independently curate each gene. Each curation team was led by a board-certified medical geneticist and included 2 additional members. All gene curation team members were required to have graduate degrees in human genetics (3 master’s degrees, 3 MD/PhD scientists, and 3 MD clinical geneticists). Gene curation teams worked blinded to other curation teams and utilized the recently proposed ClinGen gene curation framework.^7^ Curation team members were required to review a standard operating procedure for gene curation using this framework (https://www.clinicalgenome.org/curation-activities/gene-disease-validity/educational-and-training-materials/standard-operating-procedures/) and received a webinar presentation illustrating the application of the analytic process.

A detailed description of the ClinGen gene-disease validity classification framework has been published recently.^7^ This framework provides a systematic, evidence-based approach for assessing reported gene-disease associations. Using a semiquantitative scoring system, each gene-disease relationship is categorized into 4 clinical validity classification levels based on the sum of its accompanying evidence: definitive (12–18 points and replicated literature), strong (12–18 points), moderate (7–11 points), and limited (1–6 points).

Briefly, the evidence-based framework evaluates genetic and experimental data separately and provides a scoring metric based on the level of evidence provided in the published literature for the gene. Genetic evidence scores were weighted according to the design and quality of the genetics study. For example, genes implicated in studies with familial data, variant-disease segregation, and significant logarithm of the odds scores receive a greater assigned score than genes implicated through candidate gene approaches with small cohort sizes.

Experimental evidence scores were based on the interpretation and phenotypic relevance of in vitro assays assessing functional alterations of the disease-implicated gene variants, as well as model organism or rescue studies as proposed by MacArthur et al.^8^

Details of this scoring matrix and a template spreadsheet can be accessed online^7^: https://www.clinicalgenome.org/working-groups/gene-curation/projects-initiatives/gene-disease-clinical-validity-scoring-matrix/.

A clinical domain expert panel, consisting of 9 additional individuals with collectively dozens of years of experience in the clinical care or research in the field of BrS, was tasked with performing a final evaluation and classification on a gene-by-gene basis. These panel members had the option of modifying the classification of each gene (upgrade, no change, and downgrade) based on their collective experience and independent assessment of the medical literature and scientific evidence after review of curator team summaries. Each individual on the panel independently reviewed data for each gene, and together the panel discussed classifications for each gene in teleconference and face-to-face meetings (Figure I in the online-only Data Supplement). In the end, the chair of the panel requested that each individual provide a confidential vote on a final classification for each gene.

## Results

### Identifying Brugada Genes for Curation

**Figure 1.**
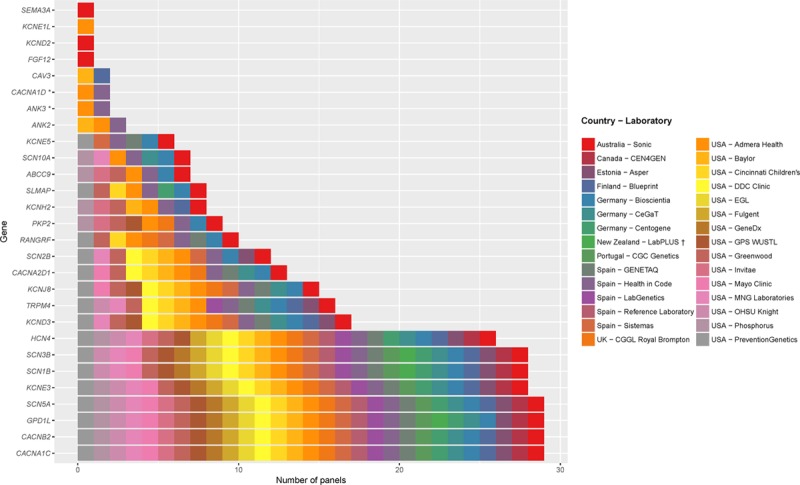
**Genes included on clinical gene test panels for Brugada syndrome**. Thirty multigene panels exclusively offered for Brugada syndrome were analyzed. The *x* axis shows the number of panels including each gene. Bars are color coded based on the labs offering the test. A list of labs is provided in the legend to the right of the graph. *The 2 Brugada syndrome panels testing for *ANK3* and *CACNA1D* have marked them as “candidate genes with no evidence, but likely to be related to the phenotype.” 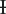
LabPLUS offers a panel for BrS types 2, 5, 7, which does not include *SCN5A*, *CACNA1C*, and *CACNB2*.

Using a PubMed search (search terms: gene [from Bookshelf] for PubMed [search Brugada syndrome gene]), we identified 23 genes reported to be associated with BrS (Table 1). As of September 2017, we identified 30 accredited laboratories offering specific BrS multigene panels, 15 in the United States, 15 internationally (NCBI Genetic Testing Registry: https://www.ncbi.nlm.nih.gov/gtr/ queried June 29, 2017; GeneTests website, https://www.genetests.org/disorders/?disid=33991, accessed September 9, 2017). These panels varied in the number of genes tested (range 3–23 genes, median 11 genes per panel). Only 4 genes were present on all testing panels (*SCN5A*, *GPD1L*, *CACNA1C*, and *CACNB2*). Of the 23 genes, 21 were reported in the literature in ≥2 peer-reviewed publications and were present on ≥3 BrS clinical genetic testing panels (range 3–29 panels, median 15 panels per gene) (Figure 1). These 21 genes were selected for gene curation.

**Table 1.**
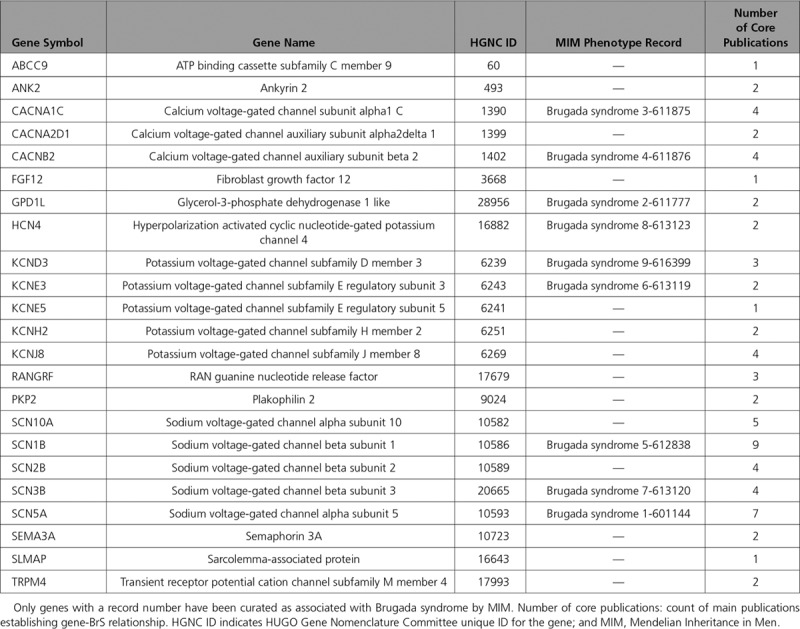
Reported Genes for Brugada Syndrome

### Gene Curation

Over 6 months, 3 gene curation teams independently reviewed the published literature for each gene and applied the analytic framework, spending 318·5 hours (14·5±5·9 hours per gene, mean±SD). A total of 130 publications were reviewed and logged by curation teams (average 7 publications per gene, range 2–19). A complete list of publications reviewed for each gene can be found in the online-only Data Supplement.

**Figure 2.**
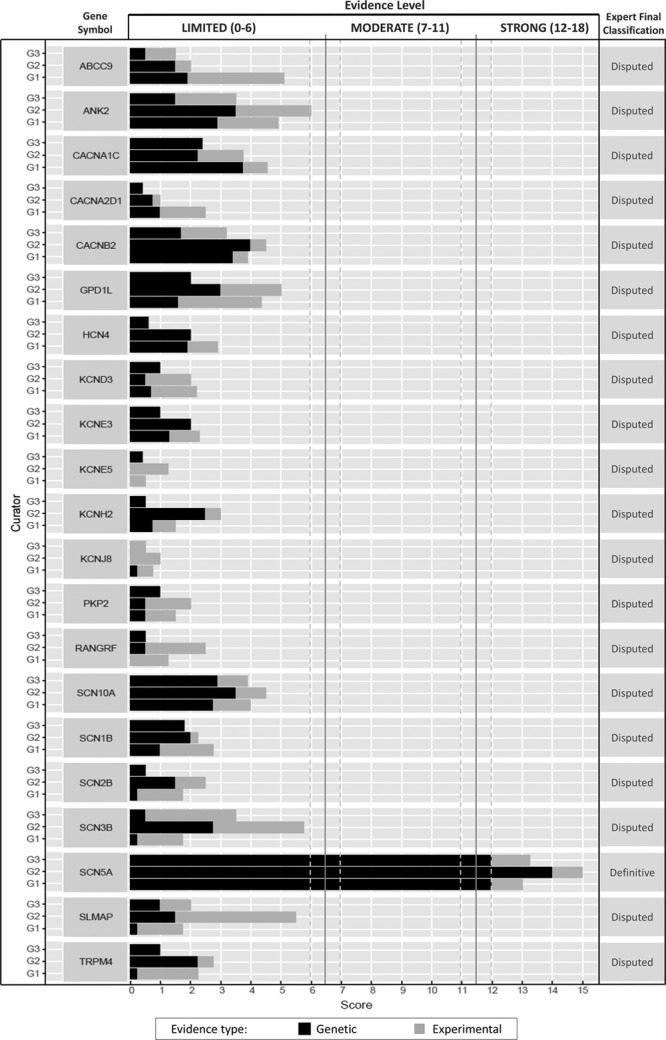
**Clinical validity classifications and matrix scores for Brugada syndrome gene associations.** Of the 21 Brugada syndrome genes (y-axis) curated, only *SCN5A* reached definitive classification; all other genes were classified as limited. Each bar represents scores from a single curator group (G) (G1, G2, G3).

Figure 2 summarizes the clinical validity classifications and semiquantitative scores for genetic and experimental evidence from curation teams for each gene. There was complete concordance among the 3 curation teams in the clinical validity classifications of all 21 genes. *SCN5A* was the only gene that reached the definitive evidence tier for BrS. All other genes (20 of 21) were classified as limited evidence by the curator teams.

The clinical domain expert panel, although agreeing with the application of the scoring template by curator teams, reclassified all 20 limited evidence genes as disputed, concluding that currently published literature is not sufficient to assert causality for BrS for any of these 20 genes. Consensus of the expert panel was unanimous (voted 9-0) for reclassification based on specific issues related to the methodology of genetic studies, the lack of supportive statistical evidence, the absence of genetically altered animal models recapitulating disease, and the uncertain interpretation of in vitro experimental data as related to the disease phenotype. These concepts are discussed in detail in the Discussion section. Summary tables on a gene by gene basis are available in the online-only Data Supplement (scores).

### Query of ClinVar Submissions

**Figure 3.**
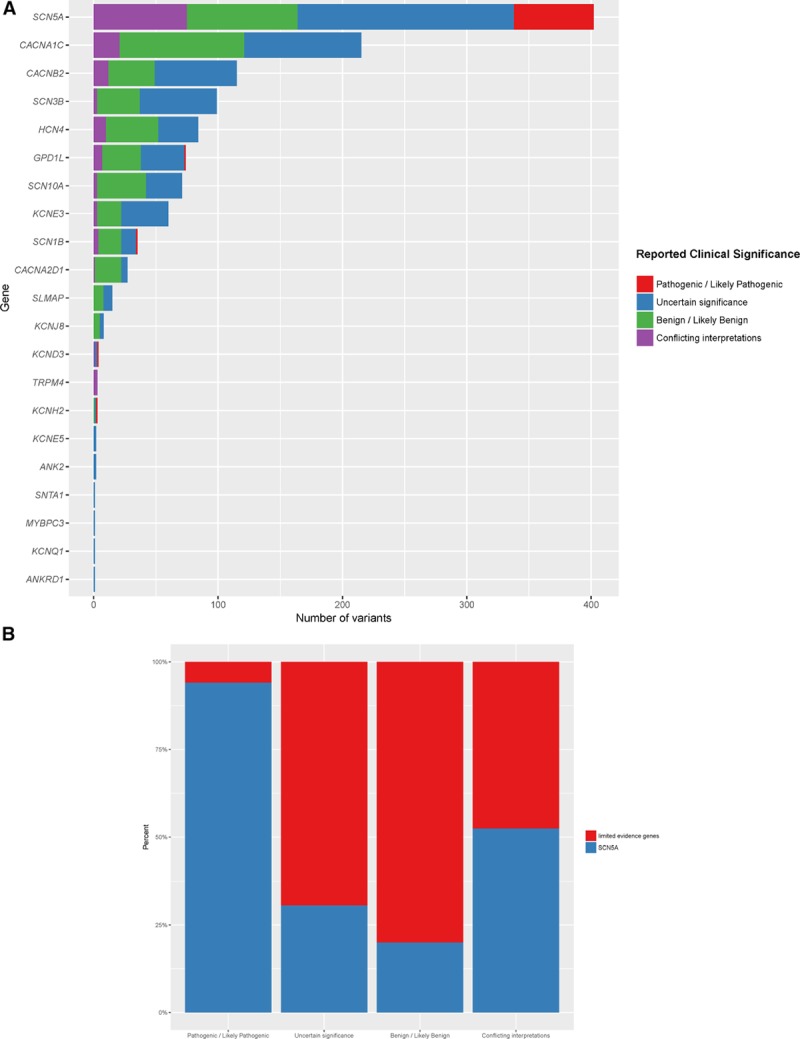
**ClinVar variants for Brugada syndrome by gene and clinical interpretation. A**, All variants submitted to ClinVar (http://clinvar.com/) by clinical labs for Brugada syndrome have been plotted (N=1223, excluding 182 variants that were literature only or research). Genes are listed along the y axis, whereas the x axis shows the count of variants for each gene. Bars are color coded based on the clinical classification of variants (see legend). **B**, The relative proportion of submitted variant interpretation classifications for SCN5A and Limited evidence genes.

To evaluate the potential impact of available testing panels on gene variant interpretations, we analyzed the number of ClinVar submissions for BrS by clinical testing laboratories. Our query (Brugada syndrome as [disease/phenotype] filters: clinical testing [queried November 1, 2017], ie, excluding research and literature only submissions) returned 1223 variations for BrS in 21 different genes, 33% in *SCN5A* (Figure 3A). Of variants classified as pathogenic and likely pathogenic, 6% were in the 20 disputed evidence genes, whereas the remainder were in *SCN5A*. In total, disputed evidence genes most commonly had submitted variants classified as uncertain significance or with conflicting interpretations (56% or 420/747 of submitted variants) (Figure 3B).

## Discussion

In this study, we performed an evidence-based curation of 21 genes reported as single gene causes for BrS that are routinely tested in patients by accredited laboratories’ clinical genetic testing panels for this sudden arrhythmic death condition. Remarkably, only 1 (*SCN5A*) of 21 genes was classified as a definitive evidence gene for BrS. All other genes (20 of 21) received a final classification of disputed with regard to assertions toward causality for BrS by a clinical domain expert panel.

The findings of this study challenge the inclusion of 20 genes, out of 21, currently offered for clinical genetic testing for BrS. All 3 curator teams and the clinical domain expert panel agreed that only *SCN5A* has sufficient evidence for causality in BrS to warrant inclusion on clinical genetic testing panels and that current evidence does not support causality or clinical testing of the 20 additional curated genes. The expert panel cited the following facts that alone or in combination compelled the conclusion of disputed gene classification: (1) familial or segregation data of affected cases with rare variants was insufficient to support causality for the gene in most studies; when sufficient data for familial linkage to a genomic region were present, comprehensive sequencing data of all rare variants shared among affected individuals within the shared genomic region were not provided; (2) reported genes were implied to have causality for BrS on the basis of rare gene variants identified in BrS cohorts, an observation that is insufficient to claim causality in light of the now known observation of frequent, benign rare genetic variations in healthy human populations common to most genes; (3) the frequency of the reported rare gene variants in BrS cases leading to claims of causality were subsequently determined to be grossly underestimated with observed frequencies in the general population that are incompatible with disease causation based on disease prevalence; (4) supportive functional data were limited to in vitro experiments, suggesting plausible molecular mechanism but without phenotypic recapitulation and in the absence of validated assays proven to distinguish disease-causing rare genetic variants from benign rare genetic variants; and (5) existing data do not demonstrate statistical evidence of an excess of rare genetic variants for the gene in BrS cases versus healthy controls.

Of the 20 genes classified as disputed, 19 of 20 were originally reported in preconceived candidate gene studies on the basis of biological plausibility, in contrast to an a priori familial linkage or other unbiased genome-wide methodology, approaches advocated for gene discovery.^8^ Seminal manuscripts for 13 of the 19 genes reported rare variants in only sporadic cases with no segregation data or evidence of rare variant excess compared with control subjects (*ABCC9*, *ANK2*, *CACNA2D1*, *HCN4*, *KCND3*, *KCNH2*, *KCNJ8*, *RANGRF*, *SCN1B*, *SCN2B*, *SCN3B*, *SLMAP*, *TRPM4*). Three genes were reported with limited segregation in only 2 individuals in 2 generations (*CACNA1C*, *KCNE5*, *and SCN10A*). Only 3 genes (*CACNB2*, *KCNE3*, *PKP2*) were reported to have >2 segregations within a family.^9–11^ Although the original description of a family harboring a *CACNB2* variant identified via a candidate gene approach indicated 6 segregations,^9^ our curators noted a reported affected individual who did not carry the reported variant in *CACNB2*. Further independent reports were not identified with sufficient genetic or statistical evidence to warrant classification beyond the disputed evidence tier for this gene. Similarly, 4 and 3 segregations were reported in original manuscripts using preconceived candidate gene approaches implicating *KCNE3* and *PKP2*, respectively, but without sufficient statistical data, additional families or supporting evidence in the literature.^10,11^

The *SCN5A* gene was the only gene, among the 21 curated, to be classified as definitive by all 3 curation teams. It is interesting to note that the original manuscript reporting this gene’s association with BrS was also a candidate gene study in small pedigrees.^12^ However, subsequent papers reported larger pedigrees with segregation and sufficient statistical evidence supporting gene causality.^13,14^ In addition, curators and the expert panel cited the dense literature reporting protein-truncating variants in this gene segregating with phenotype, and a published rare variant (minor allele frequency <0.001) burden analysis of genes reported to be associated with BrS, which identified a significant excess of *SCN5A* variants in BrS cases compared with healthy controls (20·4% versus 2·4%, *P*<1·4×10^–7^).^15^ In the same analysis, the authors evaluated 18 of the 20 disputed evidence genes and did not find any significant enrichment of rare variants in BrS cases.^15^

Notably, the *GPD1L* gene received limited evidence classification by all 3 curator teams and a final classification of disputed, despite its identification through the use of genetic linkage. Although genes identified with significant segregation and apparent linkage to phenotype may receive higher scoring in the application of the evidence-based template, the assigned scoring depends on whether comprehensive sequencing of all genes in the linked genomic region was performed versus selected screening of only specific genes. In the case of *GPD1L*, curators and the expert panel noted the extensive size of the linked genomic region, the select sequencing of only a limited number of genes within this large region (≈ 24 million base pairs) with lack of comprehensive sequencing of the region to assess for alternative gene variants, the observation that the reported variant in this gene is now recognized to be present in 1/5000 individuals in public databases (http://gnomad.broadinstitute.org/gene/ENSG00000152642), and the absence of any subsequent familial descriptions since the seminal publication in 2002.^16,17^

It is only recently appreciated that many benign variants can be extremely rare in a population, and therefore a finding of rare variation in a gene in patients with a genetic condition is far from sufficient evidence to assert causality. Thirteen of the 20 disputed evidence genes were published earlier than 2013 before the availability of large public databases indicating gene variant frequencies in thousands of individuals, where there is no reason to anticipate overrepresentation of this particular disorder with a 1 in 2000 prevalence. In the absence of large databases to compare variant frequencies found in disease cases, early gene-disease reports typically evaluated a small cohort of controls (100–1000 samples) to decide on variant rarity. Two curated genes in this study were originally implicated on the basis of variants suggested to be rare in the original studies, only to be subsequently noted to have heterozygosity frequency in public databases greater than or equal to the prevalence of BrS (*KCNJ8* p.Ser422Leu, 1/250; *SCN3B* p.Leu10Pro, p.Val110Ile, both 1/2500). This issue is further highlighted in a publication from Risgaard et al^18^ assessing the prevalence of previously concluded disease-causing mutations from original publications implicating 12 BrS genes. They found that of ≈4000 individual exomes made publicly available in 2012 from the National Heart, Lung, and Blood Institute GO Exome Sequencing Project, 1 in 23 individuals carried an originally reported putative BrS mutation. Despite classification as a definitive evidence gene, an estimated 10% to 20% of early reported putative mutations in *SCN5A* may have been erroneously classified, highlighting the challenges of variant interpretation for even definitive genes in the absence of large databases.^19^

Last, the reported BrS genes curated in this study typically provided in vitro functional data to suggest a plausible molecular mechanism for disease caused by rare variants. However, an altered in vitro function demonstrated in noncardiac, immortalized cell lines in the absence of phenotypic recapitulation in an intact animal is not synonymous with proof of disease causation. Further, none of the experimental in vitro methodologies utilized for these 20 disputed genes has been validated to distinguish the function of benign rare variants known to exist in healthy populations from the reported disease-causing rare variants. To illustrate this issue, the reported *KCNJ8* variant p.Ser422Leu originally implicated as disease-causing for BrS was reported to have a 2-fold gain of function of ion current in vitro compared with wild type despite its presence in 1/250 individuals in the general population, a frequency far too common to be a cause of BrS.^20,21^ Similarly, significant functional differences were reported for variants reported in *SCN3B* as a cause for BrS, despite the frequent presence of these same variants in presumably healthy populations.^22,23^ In the context of heart rhythm and electrocardiographic features in humans, it would be expected that both rare and common gene variants confer functional differences explaining the large range of normal variation in heart rates, QRS and QT parameters. Indeed, it is recognized that common gene variations (minor allele frequency >0.5% to 20%) in disease-causing channelopathy genes such as *KCNH2* and *SCN5A* may provoke functional alterations in vitro similar to putative mutations in those same genes and yet not lead to an associated phenotype.^24,25^

Given the results of this gene curation effort, it is important to thoughtfully consider why accredited laboratories include genes on BrS testing panels that do not have sufficient evidence for disease causality. Foremost, accreditation bodies do not require laboratories to justify the inclusion of genes on panels for clinical testing. It is also possible that the increasingly competitive marketplace in laboratory genetics motivated a more-genes-is-better approach, leading to rapidly expanding gene panels. The practical implications of including these disputed evidence genes on testing panels in clinical care are potentially harmful. Physicians and genetic counselors may trust that the inclusion of genes on disease panels by accredited laboratories implies that they have valid associations with diseases. However, the testing of genes with insufficient evidence for causality creates unnecessary challenges for variant interpretation, particularly for predicted loss-of-function variants that may be incorrectly assumed to be pathogenic and enhances the possibility of false-positive interpretations, a scenario that may lead to inappropriate risk prediction in family members, unnecessary clinical testing, prophylactic therapy, and significant distress within a family. This is especially concerning for age-related genetic diseases with incomplete penetrance, such as BrS, where genetic observations may be unduly persuasive toward diagnostic conclusions. These concerns are not merely hypothetical. Our query of ClinVar indicated that of submitted variants classified as pathogenic or likely pathogenic for BrS, 6% represented variants in disputed genes. In addition, >50% of the variants submitted in these disputed genes were classified as uncertain significance or had conflicting interpretations. The ramifications of these interpretations for variants in genes with insufficient evidence to support causality for this sudden death condition are concerning and may have led to inappropriate care in some families.

The conclusion of the Human Genome Project in 2003 was met with great enthusiasm in all fields of medicine and led to the rapid reporting of thousands of genes over the last decade as being disease-causing. Our study highlights the increasing threshold now necessary to conclude gene-disease causality in light of the evolving knowledge of natural genetic variations in the population and the need for cautious interpretation of functional assays that do not recapitulate the disease phenotype and are not validated to distinguish rare benign from rare disease-causing variants. Although the majority of reported gene-disease associations in our study currently lack sufficient evidence to warrant their inclusion for clinical genetic testing to direct care for BrS patients and families, many of these genes should remain in the realm of research, and some of these genes could ultimately gain clinical validity in future gene curation efforts. However, further research aimed to promote disputed genes or invoke novel single-gene causes for disease should provide genetic evidence using an unbiased analysis of genomic data with supportive statistical evidence. These may include studies of large families or kindreds, which are unfortunately uncommon in BrS. Alternatively, large case-control cohorts demonstrating a statistically significant excess of rare variants in a gene of interest among cases could satisfy strong genetic evidence. In light of the common observation of sporadic, nonfamilial cases of BrS, oligogenic inheritance may play a significant role and create a more challenging genetic landscape to study.

Successful implementation of precision medicine requires that the inclusion of genes on diagnostic testing panels accurately reflects clinically valid gene-disease associations. In the absence of clinical validity of tested genes, unnecessary, costly, and potentially harmful clinical tests or interventions may be ordered and used in the care and clinical decision making for a patient or family. If precision medicine is to optimize the care of patients and families, it is essential that practitioners, counselors, and diagnostic laboratories come together to ensure the most appropriate inclusion of genes for diagnostic testing and subsequent interpretation. Our findings warrant a systematic, evidence-based approach to assess the validity of reported gene-disease associations before use in patient care.

## Acknowledgments

M.H.G. designed the study. S.U. and M.H.G. conducted an initial literature search and determined genes to be curated. S.M.H., G.C., R.J., E.L., S.M.J., M.S., C.F.M., S.B., and R.H.K. performed the evidence-based gene biocuration. S.M.H. summarized the data, conducted the statistical analyses, and generated figures. J.G., M.C., A.C.S., V.N., J.S.W., R.E.H., M.J.A., A.A.M.W., and M.H.G. were on the clinical eomain expert panel and reviewed biocuration data and analyses. M.H.G. wrote the article. All authors reviewed and edited the article. The authors thank Jennifer Goldstein, PhD, C. Lisa Kurtz, PhD, and Roozbeh Manshaei, PhD, for their assistance.

## Sources of Funding

The US National Human Genome Research Institute partially funded this study. Funding agency members had no role in the study design or collection, analysis, and interpretation of the data or writing of the manuscript. The corresponding author had full access to all the data in the study and had final responsibility for the decision to submit for publication. The Clinical Genome Resource Consortium is funded by the National Human Genome Research Institute (U41 HG006834, U01 HG007436). Dr Ware is supported by Wellcome Trust (107469/Z15/Z) and the British Heart Foundation. Dr Wilde is supported by The Netherlands Cardiovascular Research Initiative (Predict). Dr Gollob is supported by the Heart and Stroke Foundation Mid-Career Scientist Award (MC7449) and the Peter Munk Research Chair in Cardiovascular Molecular Medicine (Toronto General Hospital, University of Toronto).

## Disclosures

None.

## Supplementary Material
