# Supplementary material for: Reappraisal of Reported Genes for Sudden Arrhythmic Death: Evidence-Based Evaluation of Gene Validity for Brugada Syndrome
Source: Circulation. 2018 Sep 17;138(12):1195–205. doi: 10.1161/CIRCULATIONAHA.118.035070 (PMC6147087; doi:10.1161/CIRCULATIONAHA.118.035070)
Supplement: Supplementary file 1 [file cir-138-1195-s001.pdf]

Supplemental Material

Supplement to:

Reappraisal of Reported Genes for Sudden Arrhythmic Death: An Evidence-Based Evaluation of Gene Validity for Brugada Syndrome.

Detailed gene by gene scoring files are available in an accompanying excel file to this manuscript.

NIH-Funded ClinGen Clinical Domain Expert Panel:

Michael H. Gollob, M.D. (Chair)
Toronto General Hospital, University of Toronto, Toronto, Canada

Michael J. Ackerman, M.D., Ph.D.
Mayo Clinic, Rochester, USA

Melanie Care, M.Sc.
Toronto General Hospital, University of Toronto, Toronto, Canada

John Garcia, Ph.D.
Invitae Corp, San Francisco, USA

Ray E. Hershberger, M.D.
Ohio State University, Columbus, USA

Valeria Novelli, Ph.D.
Catholic University of the Sacred Heart, Rome, Italy

Amy C. Sturm, M.Sc.
Geisinger Health System Genomic Medicine Institute, Danville, USA

James S. Ware, Ph.D.
Imperial College London, London, UK

Arthur A. M. Wilde, M.D., Ph.D.
Amsterdam Academic Medical Center, Amsterdam, The Netherlands

Bio-curators:

Sarah Bowdin, M.D., M.Sc.
The Hospital for Sick Children, Toronto, Canada

Gregory Costain, M.D., Ph.D.
The Hospital for Sick Children, Toronto, Canada

S. Mohsen Hosseini, M.D., M.Sc., Ph.D.
The Hospital for Sick Children, Toronto, Canada

Seema M. Jamal, M.Sc.
The Hospital for Sick Children, Toronto, Canada

Rebekah Jobling, M.D.
The Hospital for Sick Children, Toronto, Canada

Raymond H. Kim, M.D., Ph.D.
The Fred A. Litwin Family Centre in Genetic Medicine, University of Toronto, Toronto, Canada

Eriskay Liston, M.Sc.
The Hospital for Sick Children, Toronto, Canada

Chantal F. Morel, M.D.
The Fred A. Litwin Family Centre in Genetic Medicine, University of Toronto, Toronto, Canada

Marta Szybowska, M.Sc.
The Fred A. Litwin Family Centre in Genetic Medicine, University of Toronto, Toronto, Canada

Figure 1
BrS Gene Curation Process

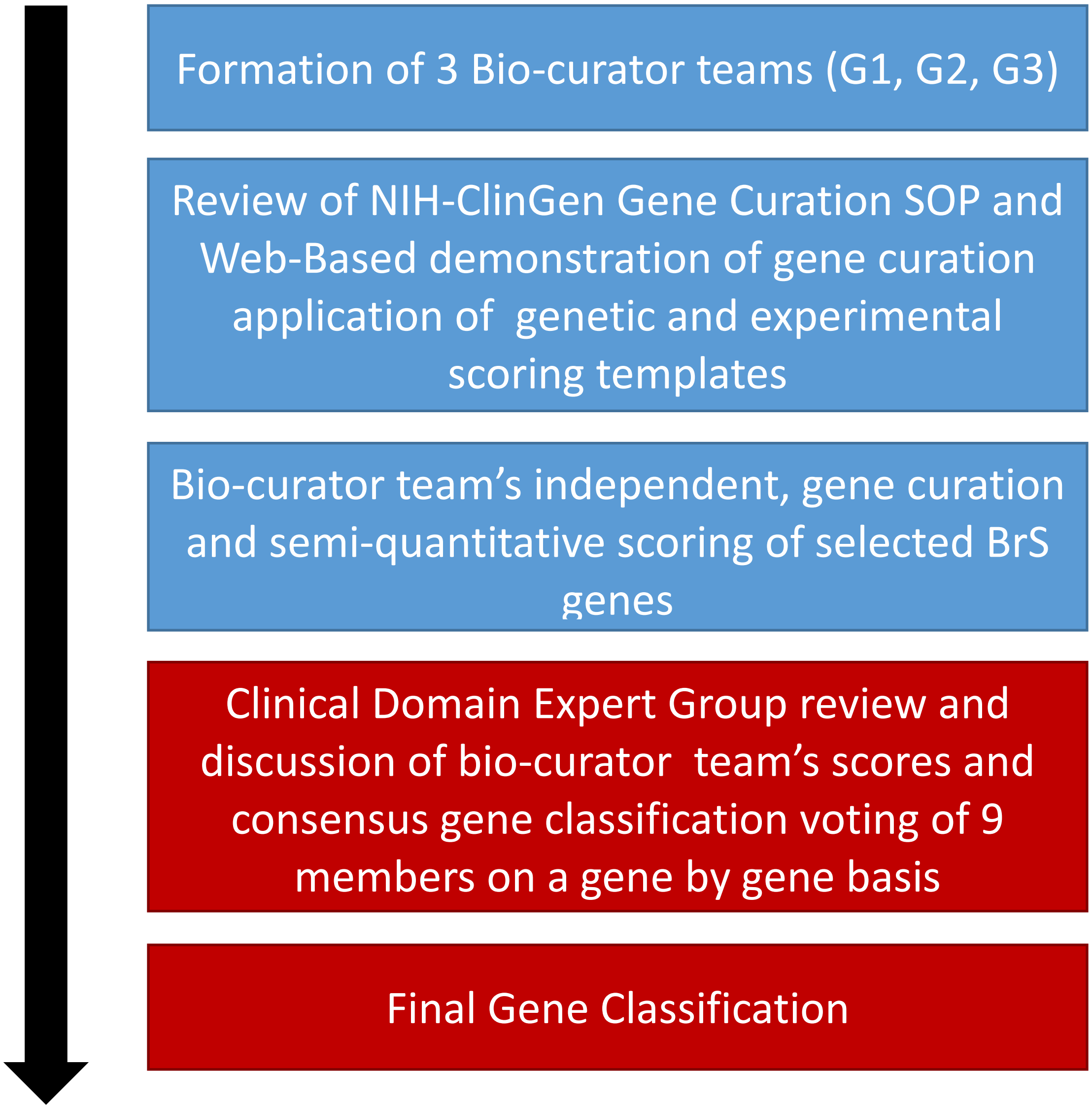

List of Publications Reviewed for Brugada Syndrome Gene Curation

Multiple Genes

1. Antzelevitch C, Pollevick GD, Cordeiro JM, Casis O, Sanguinetti MC, Aizawa Y, Guerchicoff A, Pfeiffer R, Oliva A, Wollnik B, Gelber P, Bonaros EP, Jr., Burashnikov E, Wu Y, Sargent JD, Schickel S, Oberheiden R, Bhatia A, Hsu LF, Haissaguerre M, Schimpf R, Borggrefe M and Wolpert C. Loss-of-function mutations in the cardiac calcium channel underlie a new clinical entity characterized by ST-segment elevation, short QT intervals, and sudden cardiac death. *Circulation*. 2007;115:442-449.

2. Koopmann TT, Beekman L, Alders M, Meregalli PG, Mannens MM, Moorman AF, Wilde AA and Bezzina CR. Exclusion of multiple candidate genes and large genomic rearrangements in SCN5A in a Dutch Brugada syndrome cohort. *Heart Rhythm*. 2007;4:752-755.

3. Burashnikov E, Pfeiffer R, Barajas-Martinez H, Delpon E, Hu D, Desai M, Borggrefe M, Haissaguerre M, Kanter R, Pollevick GD, Guerchicoff A, Laino R, Marieb M, Nademanee K, Nam GB, Robles R, Schimpf R, Stapleton DD, Viskin S, Winters S, Wolpert C, Zimmern S, Veltmann C and Antzelevitch C. Mutations in the cardiac L-type calcium channel associated with inherited J-wave syndromes and sudden cardiac death. *Heart Rhythm*. 2010;7:1872-1882.

4. Mishra S, Undrovinas NA, Maltsev VA, Reznikov V, Sabbah HN and Undrovinas A. Post-transcriptional silencing of SCN1B and SCN2B genes modulates late sodium current in cardiac myocytes from normal dogs and dogs with chronic heart failure. *Am J Physiol Heart Circ Physiol*. 2011;301:H1596-1605.

5. Crotti L, Marcou CA, Tester DJ, Castelletti S, Giudicessi JR, Torchio M, Medeiros-Domingo A, Simone S, Will ML, Dagradi F, Schwartz PJ and Ackerman MJ. Spectrum and prevalence of mutations involving BrS1-through BrS12-susceptibility genes in a cohort of unrelated patients referred for Brugada syndrome genetic testing: implications for genetic testing. *J Am Coll Cardiol*. 2012;60:1410-1418.

6. Duthoit G, Fressart V, Hidden-Lucet F, Simon F, Kattygnarath D, Charron P, Himbert C, Aouate P, Guicheney P, Lecarpentier Y, Frank R and Hebert JL. Brugada ECG pattern: a physiopathological prospective study based on clinical, electrophysiological, angiographic, and genetic findings. *Front Physiol.* 2012;3:1-16.

7. Holst AG, Saber S, Houshmand M, Zaklyazminskaya EV, Wang Y, Jensen HK, Refsgaard L, Haunso S, Svendsen JH, Olesen MS and Tfelt-Hansen J. Sodium current and potassium transient outward current genes in Brugada syndrome: screening and bioinformatics. *Can J Cardiol*. 2012;28:196-200.

8. Risgaard B, Jabbari R, Refsgaard L, Holst AG, Haunso S, Sadjadieh A, Winkel BG, Olesen MS and Tfelt-Hansen J. High prevalence of genetic variants previously associated with Brugada syndrome in new exome data. *Clin Genet*. 2013;84:489-495.

9. Allegue C, Coll M, Mates J, Campuzano O, Iglesias A, Sobrino B, Brion M, Amigo J, Carracedo A, Brugada P, Brugada J and Brugada R. Genetic Analysis of Arrhythmogenic Diseases in the Era of NGS: The Complexity of Clinical Decision-Making in Brugada Syndrome. *PloS one*. 2015;10:1-21.

10. Hasdemir C, Payzin S, Kocabas U, Sahin H, Yildirim N, Alp A, Aydin M, Pfeiffer R, Burashnikov E, Wu Y and Antzelevitch C. High prevalence of concealed Brugada syndrome in patients with atrioventricular nodal reentrant tachycardia. *Heart Rhythm*. 2015;12:1584-1594.

11. Le Scouarnec S, Karakachoff M, Gourraud JB, Lindenbaum P, Bonnaud S, Portero V, Duboscq-Bidot L, Daumy X, Simonet F, Teusan R, Baron E, Violleau J, Persyn E, Bellanger L, Barc J, Chatel S, Martins R, Mabo P, Sacher F, Haissaguerre M, Kyndt F, Schmitt S, Bezieau S, Le Marec H, Dina C, Schott JJ, Probst V and Redon R. Testing the burden of rare variation in arrhythmia-susceptibility genes provides new insights into molecular diagnosis for Brugada syndrome. *Hum Mol Genet*. 2015;24:2757-2763.

12. Peeters U, Scornik F, Riuro H, Perez G, Komurcu-Bayrak E, Van Malderen S, Pappaert G, Tarradas A, Pagans S, Daneels D, Breckpot K, Brugada P, Bonduelle M, Brugada R and Van Dooren S. Contribution of Cardiac Sodium Channel beta-Subunit Variants to Brugada Syndrome. *Circ J.* 2015;79:2118-2129.

13. Perez-Serra A, Mademont-Soler I, Riuro H, Pico F, Coll M, Iglesias A, Pagans S, Sarquella-Brugada G, Berne P, Benito B, Brugada J, Porres JM, Lopez Zea M, Castro-Urda V, Fernandez-Lozano I and Brugada R. Comprehensive Genetic Characterization of a Spanish Brugada Syndrome Cohort. *PloS one*. 2015;10:1-15.

14. Ghouse J, Have CT, Skov MW, Andreasen L, Ahlberg G, Nielsen JB, Skaaby T, Olesen SP, Grarup N, Linneberg A, Pedersen O, Vestergaard H, Haunso S, Svendsen JH, Hansen T, Kanters JK and Olesen MS. Numerous Brugada syndrome-associated genetic variants have no effect on J-point elevation, syncope susceptibility, malignant cardiac arrhythmia, and all-cause mortality. *Genet Med*. 2017;19:521-528.

ABCC9

15. Chutkow WA, Simon MC, Le Beau MM and Burant CF. Cloning, tissue expression, and chromosomal localization of SUR2, the putative drug-binding subunit of cardiac, skeletal muscle, and vascular KATP channels. *Diabetes*. 1996;45:1439-1445.

16. Bienengraeber M, Alekseev AE, Abraham MR, Carrasco AJ, Moreau C, Vivaudou M, Dzeja PP and Terzic A. ATPase activity of the sulfonylurea receptor: a catalytic function for the KATP channel complex. *FASEB J*. 2000;14:1943-1952.

17. Chutkow WA, Pu J, Wheeler MT, Wada T, Makielski JC, Burant CF and McNally EM. Episodic coronary artery vasospasm and hypertension develop in the absence of Sur2 K(ATP) channels. *J Clin Invest*. 2002;110:203-208.

18. Bienengraeber M, Olson TM, Selivanov VA, Kathmann EC, O'Cochlain F, Gao F, Karger AB, Ballew JD, Hodgson DM, Zingman LV, Pang YP, Alekseev AE and Terzic A. ABCC9 mutations identified in human dilated cardiomyopathy disrupt catalytic KATP channel gating. *Nat Genet*. 2004;36:382-387.

19. Beziau DM, Barc J, O'Hara T, Le Gloan L, Amarouch MY, Solnon A, Pavin D, Lecointe S, Bouillet P, Gourraud JB, Guicheney P, Denjoy I, Redon R, Mabo P, le Marec H, Loussouarn G, Kyndt F, Schott JJ, Probst V and Baro I. Complex Brugada syndrome inheritance in a family harbouring compound SCN5A and CACNA1C mutations. *Basic Res Cardiol*. 2014;109:446.

20. Hu D, Barajas-Martinez H, Terzic A, Park S, Pfeiffer R, Burashnikov E, Wu Y, Borggrefe M, Veltmann C, Schimpf R, Cai JJ, Nam GB, Deshmukh P, Scheinman M, Preminger M, Steinberg J, Lopez-Izquierdo A, Ponce-Balbuena D, Wolpert C, Haissaguerre M, Sanchez-Chapula JA and Antzelevitch C. ABCC9 is a novel Brugada and early repolarization syndrome susceptibility gene. *Int J Cardiol*. 2014;171:431-442.

ANK2

21. Mohler PJ, Rivolta I, Napolitano C, LeMaillet G, Lambert S, Priori SG and Bennett V. Nav1.5 E1053K mutation causing Brugada syndrome blocks binding to ankyrin-G and expression of Nav1.5 on the surface of cardiomyocytes. *Proc Natl Acad Sci*. 2004;101:17533-17538.

22. Mohler PJ, Le Scouarnec S, Denjoy I, Lowe JS, Guicheney P, Caron L, Driskell IM, Schott JJ, Norris K, Leenhardt A, Kim RB, Escande D and Roden DM. Defining the cellular phenotype of "ankyrin-B syndrome" variants: human ANK2 variants associated with clinical phenotypes display a spectrum of activities in cardiomyocytes. *Circulation*. 2007;115:432-441.

23. Ichikawa M, Aiba T, Ohno S, Shigemizu D, Ozawa J, Sonoda K, Fukuyama M, Itoh H, Miyamoto Y, Tsunoda T, Makiyama T, Tanaka T, Shimizu W and Horie M. Phenotypic Variability of ANK2 Mutations in Patients With Inherited Primary Arrhythmia Syndromes. *Circ J*. 2016;80:2435-2442.

CACNA1C

24. Schultz D, Mikala G, Yatani A, Engle DB, Iles DE, Segers B, Sinke RJ, Weghuis DO, Klockner U, Wakamori M and et al. Cloning, chromosomal localization, and functional expression of the alpha 1 subunit of the L-type voltage-dependent calcium channel from normal human heart. *Proc Natl Acad Sci*. 1993;90:6228-6232.

25. Fukuyama M, Ohno S, Wang Q, Kimura H, Makiyama T, Itoh H, Ito M and Horie M. L-type calcium channel mutations in Japanese patients with inherited arrhythmias. *Circ J*. 2013;77:1799-1806.

26. Fukuyama M, Ohno S, Wang Q, Shirayama T, Itoh H and Horie M. Nonsense-mediated mRNA decay due to a CACNA1C splicing mutation in a patient with Brugada syndrome. *Heart Rhythm*. 2014;11:629-634.

27. Simms BA, Souza IA and Zamponi GW. Effect of the Brugada syndrome mutation A39V on calmodulin regulation of Cav1.2 channels. *Mol Brain*. 2014;7:34.

28. Canpolat U, Coteli C and Aytemir K. Brugada syndrome and calcium channel mutation in a patient with congenital deaf mutism. *Indian Pacing Electrophysiol*. 2017;17:16-17.

CACNA2D1

29. Fuller-Bicer GA, Varadi G, Koch SE, Ishii M, Bodi I, Kadeer N, Muth JN, Mikala G, Petrashevskaya NN, Jordan MA, Zhang SP, Qin N, Flores CM, Isaacsohn I, Varadi M, Mori Y, Jones WK and Schwartz A. Targeted disruption of the voltage-dependent calcium channel alpha2/delta-1-subunit. *Am J Physiol Heart Circ Physiol*. 2009;297:H117-124.

30. Fagerberg L, Hallstrom BM, Oksvold P, Kampf C, Djureinovic D, Odeberg J, Habuka M, Tahmasebpoor S, Danielsson A, Edlund K, Asplund A, Sjostedt E, Lundberg E, Szigyarto CA, Skogs M, Takanen JO, Berling H, Tegel H, Mulder J, Nilsson P, Schwenk JM, Lindskog C, Danielsson F, Mardinoglu A, Sivertsson A, von Feilitzen K, Forsberg M, Zwahlen M, Olsson I, Navani S, Huss M, Nielsen J, Ponten F and Uhlen M. Analysis of the human tissue-specific expression by genome-wide integration of transcriptomics and antibody-based proteomics. Mol Cell Proteomics. 2014;13:397-406.

31. Bourdin B, Shakeri B, Tetreault MP, Sauve R, Lesage S and Parent L. Functional characterization of CaValpha2delta mutations associated with sudden cardiac death. *J Biol Chem*. 2015;290:2854-2869.

CACNB2

32. Yamaguchi H, Okuda M, Mikala G, Fukasawa K and Varadi G. Cloning of the beta(2a) subunit of the voltage-dependent calcium channel from human heart: cooperative effect of alpha(2)/delta and beta(2a) on the membrane expression of the alpha(1C) subunit. *Bioch Biophys Res Comm*. 2000;267:156-163.

33. Cordeiro JM, Marieb M, Pfeiffer R, Calloe K, Burashnikov E and Antzelevitch C. Accelerated inactivation of the L-type calcium current due to a mutation in CACNB2b underlies Brugada syndrome. *Mol Cell Cardiol*.. 2009;46:695-703.

34. Kanter RJ, Pfeiffer R, Hu D, Barajas-Martinez H, Carboni MP and Antzelevitch C. Brugada-like syndrome in infancy presenting with rapid ventricular tachycardia and intraventricular conduction delay. *Circulation*. 2012;125:14-22.

GPD1L

35. Weiss R, Barmada MM, Nguyen T, Seibel JS, Cavlovich D, Kornblit CA, Angelilli A, Villanueva F, McNamara DM and London B. Clinical and molecular heterogeneity in the Brugada syndrome: a novel gene locus on chromosome 3. *Circulation*. 2002;105:707-713.

36. London B, Michalec M, Mehdi H, Zhu X, Kerchner L, Sanyal S, Viswanathan PC, Pfahnl AE, Shang LL, Madhusudanan M, Baty CJ, Lagana S, Aleong R, Gutmann R, Ackerman MJ, McNamara DM, Weiss R and Dudley SC, Jr. Mutation in glycerol-3-phosphate dehydrogenase 1 like gene (GPD1-L) decreases cardiac Na+ current and causes inherited arrhythmias. *Circulation*. 2007;116:2260-2268.

37. Makiyama T, Akao M, Haruna Y, Tsuji K, Doi T, Ohno S, Nishio Y, Kita T and Horie M. Mutation analysis of the glycerol-3 phosphate dehydrogenase-1 like (GPD1L) gene in Japanese patients with Brugada syndrome. *CircJ*. 2008;72:1705-1706.

38. Valdivia CR, Ueda K, Ackerman MJ and Makielski JC. GPD1L links redox state to cardiac excitability by PKC-dependent phosphorylation of the sodium channel SCN5A. *Am J Physiol Heart Circ Physiol*. 2009;297:H1446-452.

HCN4

39. Ueda K, Nakamura K, Hayashi T, Inagaki N, Takahashi M, Arimura T, Morita H, Higashiuesato Y, Hirano Y, Yasunami M, Takishita S, Yamashina A, Ohe T, Sunamori M, Hiraoka M and Kimura A. Functional characterization of a trafficking-defective HCN4 mutation, D553N, associated with cardiac arrhythmia. *J Biol Chem*. 2004;279:27194-27198.

40. Ueda K, Hirano Y, Higashiuesato Y, Aizawa Y, Hayashi T, Inagaki N, Tana T, Ohya Y, Takishita S, Muratani H, Hiraoka M and Kimura A. Role of HCN4 channel in preventing ventricular arrhythmia. *J Hum Genet*. 2009;54:115-121.

41. Biel S, Aquila M, Hertel B, Berthold A, Neumann T, DiFrancesco D, Moroni A, Thiel G and Kauferstein S. Mutation in S6 domain of HCN4 channel in patient with suspected Brugada syndrome modifies channel function. *Pflugers Arch*. 2016;468:1663-1671.

KCND3

42. Dilks D, Ling HP, Cockett M, Sokol P and Numann R. Cloning and expression of the human kv4.3 potassium channel. *J Neurophysiol*. 1999;81:1974-1977.

43. Giudicessi JR, Ye D, Tester DJ, Crotti L, Mugione A, Nesterenko VV, Albertson RM, Antzelevitch C, Schwartz PJ and Ackerman MJ. Transient outward current (I(to)) gain-of-function mutations in the KCND3-encoded Kv4.3 potassium channel and Brugada syndrome. *Heart Rhythm*. 2011;8:1024-1032.

44. You T, Mao W, Cai B, Li F and Xu H. Two novel Brugada syndrome-associated mutations increase KV4.3 membrane expression and function. *Int J Mol Med*. 2015;36:309-315.

KCNE3

45. Lundquist AL, Manderfield LJ, Vanoye CG, Rogers CS, Donahue BS, Chang PA, Drinkwater DC, Murray KT and George AL, Jr. Expression of multiple KCNE genes in human heart may enable variable modulation of I(Ks). *J Mol Cell Cardiol*. 2005;38:277-287.

46. Delpon E, Cordeiro JM, Nunez L, Thomsen PE, Guerchicoff A, Pollevick GD, Wu Y, Kanters JK, Larsen CT, Hofman-Bang J, Burashnikov E, Christiansen M and Antzelevitch C. Functional effects of KCNE3 mutation and its role in the development of Brugada syndrome. *Circ Arrhythm Eectrophysiol*. 2008;1:209-218.

47. Nakajima T, Wu J, Kaneko Y, Ashihara T, Ohno S, Irie T, Ding WG, Matsuura H, Kurabayashi M and Horie M. KCNE3 T4A as the genetic basis of Brugada-pattern electrocardiogram. *Circ J*. 2012;76:2763-2772.

KCNE5

48. Piccini M, Vitelli F, Seri M, Galietta LJ, Moran O, Bulfone A, Banfi S, Pober B and Renieri A. KCNE1-like gene is deleted in AMME contiguous gene syndrome: identification and characterization of the human and mouse homologs. *Genomics*. 1999;60:251-257.

49. Ohno S, Zankov DP, Ding WG, Itoh H, Makiyama T, Doi T, Shizuta S, Hattori T, Miyamoto A, Naiki N, Hancox JC, Matsuura H and Horie M. KCNE5 (KCNE1L) variants are novel modulators of Brugada syndrome and idiopathic ventricular fibrillation. *Circ Arrhythm Electrophysiol*. 2011;4:352-361.

50. Abbott GW. KCNE4 and KCNE5: K(+) channel regulation and cardiac arrhythmogenesis. *Gene*. 2016;593:249-260.

KCNH2

51. Lees-Miller JP, Guo J, Somers JR, Roach DE, Sheldon RS, Rancourt DE and Duff HJ. Selective knockout of mouse ERG1 B potassium channel eliminates I(Kr) in adult ventricular myocytes and elicits episodes of abrupt sinus bradycardia. *Mol Cell Biol*. 2003;23:1856-1862.

52. Verkerk AO, Wilders R, Schulze-Bahr E, Beekman L, Bhuiyan ZA, Bertrand J, Eckardt L, Lin D, Borggrefe M, Breithardt G, Mannens MM, Tan HL, Wilde AA and Bezzina CR. Role of sequence variations in the human ether-a-go-go-related gene (HERG, KCNH2) in the Brugada syndrome. *Cardiovasc Res*. 2005;68:441-453.

53. Chung SK, MacCormick JM, McCulley CH, Crawford J, Eddy CA, Mitchell EA, Shelling AN, French JK, Skinner JR and Rees MI. Long QT and Brugada syndrome gene mutations in New Zealand. *Heart Rhythm*. 2007;4:1306-1314.

54. Itoh H, Sakaguchi T, Ashihara T, Ding WG, Nagaoka I, Oka Y, Nakazawa Y, Yao T, Jo H, Ito M, Nakamura K, Ohe T, Matsuura H and Horie M. A novel KCNH2 mutation as a modifier for short QT interval. *Int J Cardiol*. 2009;137:83-85.

55. Amin AS, Klemens CA, Verkerk AO, Meregalli PG, Asghari-Roodsari A, de Bakker JM, January CT, Wilde AA and Tan HL. Fever-triggered ventricular arrhythmias in Brugada syndrome and type 2 long-QT syndrome. *Neth Heart J*. 2010;18:165-169.

56. Wilders R and Verkerk AO. Role of the R1135H KCNH2 mutation in Brugada syndrome. *Int J Cardiol*. 2010;144:149-151.

57. Gianulis EC and Trudeau MC. Rescue of aberrant gating by a genetically encoded PAS (Per-Arnt-Sim) domain in several long QT syndrome mutant human ether-a-go-go-related gene potassium channels. *J Biol Chem*. 2011;286:22160-22169.

58. Wang Q, Ohno S, Ding WG, Fukuyama M, Miyamoto A, Itoh H, Makiyama T, Wu J, Bai J, Hasegawa K, Shinohara T, Takahashi N, Shimizu A, Matsuura H and Horie M. Gain-of-function KCNH2 mutations in patients with Brugada syndrome. *J Cardiovasc Electrophysiol*. 2014;25:522-530.

59. Saber S, Amarouch MY, Fazelifar AF, Haghjoo M, Emkanjoo Z, Alizadeh A, Houshmand M, Gavrilenko AV, Abriel H and Zaklyazminskaya EV. Complex genetic background in a large family with Brugada syndrome. *Physiol Rep*. 2015;2013.

KCNJ8

60. Miki T, Suzuki M, Shibasaki T, Uemura H, Sato T, Yamaguchi K, Koseki H, Iwanaga T, Nakaya H and Seino S. Mouse model of Prinzmetal angina by disruption of the inward rectifier Kir6.1. *Nat Med*. 2002;8:466-472.

61. Haissaguerre M, Chatel S, Sacher F, Weerasooriya R, Probst V, Loussouarn G, Horlitz M, Liersch R, Schulze-Bahr E, Wilde A, Kaab S, Koster J, Rudy Y, Le Marec H and Schott JJ. Ventricular fibrillation with prominent early repolarization associated with a rare variant of KCNJ8/KATP channel. *J Cardiovasc Electrophysiol*. 2009;20:93-98.

62. Medeiros-Domingo A, Tan BH, Crotti L, Tester DJ, Eckhardt L, Cuoretti A, Kroboth SL, Song C, Zhou Q, Kopp D, Schwartz PJ, Makielski JC and Ackerman MJ. Gain-of-function mutation S422L in the KCNJ8-encoded cardiac K(ATP) channel Kir6.1 as a pathogenic substrate for J-wave syndromes. *Heart Rhythm*. 2010;7:1466-1471.

63. Barajas-Martinez H, Hu D, Ferrer T, Onetti CG, Wu Y, Burashnikov E, Boyle M, Surman T, Urrutia J, Veltmann C, Schimpf R, Borggrefe M, Wolpert C, Ibrahim BB, Sanchez-Chapula JA, Winters S, Haissaguerre M and Antzelevitch C. Molecular genetic and functional association of Brugada and early repolarization syndromes with S422L missense mutation in KCNJ8. *Heart Rhythm*. 2012;9:548-555.

64. Nakaya H. Role of ATP-sensitive K+ channels in cardiac arrhythmias. *J Cardiovac Pharmacol Ther.*. 2014;19:237-243.

PKP2

65. Mertens C, Kuhn C and Franke WW. Plakophilins 2a and 2b: constitutive proteins of dual location in the karyoplasm and the desmosomal plaque. *J Cell Biol* 1996;135:1009-1025.

66. Grossmann KS, Grund C, Huelsken J, Behrend M, Erdmann B, Franke WW and Birchmeier W. Requirement of plakophilin 2 for heart morphogenesis and cardiac junction formation. *J Cell Biol*. 2004;167:149-160.

67. Oxford EM, Musa H, Maass K, Coombs W, Taffet SM and Delmar M. Connexin43 remodeling caused by inhibition of plakophilin-2 expression in cardiac cells. *Circ Res*. 2007;101:703-711.

68. Sato PY, Musa H, Coombs W, Guerrero-Serna G, Patino GA, Taffet SM, Isom LL and Delmar M. Loss of plakophilin-2 expression leads to decreased sodium current and slower conduction velocity in cultured cardiac myocytes. *Circ Res*. 2009;105:523-526.

69. Cerrone M, Noorman M, Lin X, Chkourko H, Liang FX, van der Nagel R, Hund T, Birchmeier W, Mohler P, van Veen TA, van Rijen HV and Delmar M. Sodium current deficit and arrhythmogenesis in a murine model of plakophilin-2 haploinsufficiency. *Cardiovasc Res*. 2012;95:460-468.

70. Cerrone M, Lin X, Zhang M, Agullo-Pascual E, Pfenniger A, Chkourko Gusky H, Novelli V, Kim C, Tirasawadichai T, Judge DP, Rothenberg E, Chen HS, Napolitano C, Priori SG and Delmar M. Missense mutations in plakophilin-2 cause sodium current deficit and associate with a Brugada syndrome phenotype. *Circulation*. 2014;129:1092-1103.

71. Peters S. Arrhythmogenic cardiomyopathy and provocable Brugada ECG in a patient caused by missense mutation in plakophilin-2. *Int J Cardiol*. 2014;173:317-318.

72. Forkmann M, Tomala J, Huo Y, Mayer J, Christoph M, Wunderlich C, Salmas J, Gaspar T and Piorkowski C. Epicardial Ventricular Tachycardia Ablation in a Patient With Brugada ECG Pattern and Mutation of PKP2 and DSP Genes. *Circ Arrhythm Electrophysiol*. 2015;8:505-507.

RANGRF

73. Wu L, Yong SL, Fan C, Ni Y, Yoo S, Zhang T, Zhang X, Obejero-Paz CA, Rho HJ, Ke T, Szafranski P, Jones SW, Chen Q and Wang QK. Identification of a new co-factor, MOG1, required for the full function of cardiac sodium channel Nav 1.5. *J Biol Chem*. 2008;283:6968-6978.

74. Kattygnarath D, Maugenre S, Neyroud N, Balse E, Ichai C, Denjoy I, Dilanian G, Martins RP, Fressart V, Berthet M, Schott JJ, Leenhardt A, Probst V, Le Marec H, Hainque B, Coulombe A, Hatem SN and Guicheney P. MOG1: a new susceptibility gene for Brugada syndrome. *Circ Cardiovasc Genet*. 2011;4:261-268.

75. Olesen MS, Jensen NF, Holst AG, Nielsen JB, Tfelt-Hansen J, Jespersen T, Sajadieh A, Haunso S, Lund JT, Calloe K, Schmitt N and Svendsen JH. A novel nonsense variant in Nav1.5 cofactor MOG1 eliminates its sodium current increasing effect and may increase the risk of arrhythmias. *Can J Cardiol*. 2011;27:523.e17-23.

76. Chakrabarti S, Wu X, Yang Z, Wu L, Yong SL, Zhang C, Hu K, Wang QK and Chen Q. MOG1 rescues defective trafficking of Na(v)1.5 mutations in Brugada syndrome and sick sinus syndrome. *Circ Arrhythm Electrophysiol*. 2013;6:392-401.

77. Campuzano O, Berne P, Selga E, Allegue C, Iglesias A, Brugada J and Brugada R. Brugada syndrome and p.E61X_RANGRF. *Cardiol J*. 2014;21:121-127.

78. Zhou J, Wang L, Zuo M, Wang X, Ahmed AS, Chen Q and Wang QK. Cardiac sodium channel regulator MOG1 regulates cardiac morphogenesis and rhythm. *Sci Rep*. 2016;6:21538.

SCN10A

79. Rabert DK, Koch BD, Ilnicka M, Obernolte RA, Naylor SL, Herman RC, Eglen RM, Hunter JC and Sangameswaran L. A tetrodotoxin-resistant voltage-gated sodium channel from human dorsal root ganglia, hPN3/SCN10A. *Pain*. 1998;78:107-14.

80. Yang T, Atack TC, Stroud DM, Zhang W, Hall L and Roden DM. Blocking Scn10a channels in heart reduces late sodium current and is antiarrhythmic. *Circ Res*. 2012;111:322-332.

81. Hu D, Barajas-Martinez H, Pfeiffer R, Dezi F, Pfeiffer J, Buch T, Betzenhauser MJ, Belardinelli L, Kahlig KM, Rajamani S, DeAntonio HJ, Myerburg RJ, Ito H, Deshmukh P, Marieb M, Nam GB, Bhatia A, Hasdemir C, Haissaguerre M, Veltmann C, Schimpf R, Borggrefe M, Viskin S and Antzelevitch C. Mutations in SCN10A are responsible for a large fraction of cases of Brugada syndrome. *J Am Coll Cardiol*. 2014;64:66-79.

82. van den Boogaard M, Smemo S, Burnicka-Turek O, Arnolds DE, van de Werken HJ, Klous P, McKean D, Muehlschlegel JD, Moosmann J, Toka O, Yang XH, Koopmann TT, Adriaens ME, Bezzina CR, de Laat W, Seidman C, Seidman JG, Christoffels VM, Nobrega MA, Barnett P and Moskowitz IP. A common genetic variant within SCN10A modulates cardiac SCN5A expression. *J Clin Invest*. 2014;124:1844-1852.

83. Behr ER, Savio-Galimberti E, Barc J, Holst AG, Petropoulou E, Prins BP, Jabbari J, Torchio M, Berthet M, Mizusawa Y, Yang T, Nannenberg EA, Dagradi F, Weeke P, Bastiaenan R, Ackerman MJ, Haunso S, Leenhardt A, Kaab S, Probst V, Redon R, Sharma S, Wilde A, Tfelt-Hansen J, Schwartz P, Roden DM, Bezzina CR, Olesen M, Darbar D, Guicheney P, Crotti L and Jamshidi Y. Role of common and rare variants in SCN10A: results from the Brugada syndrome QRS locus gene discovery collaborative study. *Cardiovasc Res*. 2015;106:520-529.

84. Fukuyama M, Ohno S, Makiyama T and Horie M. Novel SCN10A variants associated with Brugada syndrome. *Europace.* 2016;18:905-911.

85. Stroud DM, Yang T, Bersell K, Kryshtal DO, Nagao S, Shaffer C, Short L, Hall L, Atack TC, Zhang W, Knollmann BC, Baudenbacher F and Roden DM. Contrasting Nav1.8 Activity in Scn10a-/- Ventricular Myocytes and the Intact Heart. *J Am Heart Assoc*. 2016;5.

86. Zhang L, Zhou F, Huang L, Wu Q, Zheng J, Wu Y, Yin K and Cheng J. Association of common and rare variants of SCN10A gene with sudden unexplained nocturnal death syndrome in Chinese Han population. .*Int J Legal Med*. 2017;131:53-60.

SCN1B

87. Nuss HB, Chiamvimonvat N, Perez-Garcia MT, Tomaselli GF and Marban E. Functional association of the beta 1 subunit with human cardiac (hH1) and rat skeletal muscle (mu 1) sodium channel alpha subunits expressed in Xenopus oocytes. *J Gen Physiol*. 1995;106:1171-1191.

88. Qu Y, Isom LL, Westenbroek RE, Rogers JC, Tanada TN, McCormick KA, Scheuer T and Catterall WA. Modulation of cardiac Na+ channel expression in Xenopus oocytes by beta 1 subunits. *J Biol Chem*. 1995;270:25696-701.

89. Deschenes I, DiSilvestre D, Juang GJ, Wu RC, An WF and Tomaselli GF. Regulation of Kv4.3 current by KChIP2 splice variants: a component of native cardiac I(to)? *Circulation*. 2002;106:423-429.

90. Deschenes I and Tomaselli GF. Modulation of Kv4.3 current by accessory subunits. *FEBS Lett*. 2002;528:183-188.

91. Zimmer T, Bollensdorff C, Haufe V, Birch-Hirschfeld E and Benndorf K. Mouse heart Na+ channels: primary structure and function of two isoforms and alternatively spliced variants. *Am J Physiol Heart Circ Physiol*. 2002;282:H1007-1017.

92. Lopez-Santiago LF, Meadows LS, Ernst SJ, Chen C, Malhotra JD, McEwen DP, Speelman A, Noebels JL, Maier SK, Lopatin AN and Isom LL. Sodium channel Scn1b null mice exhibit prolonged QT and RR intervals. *J Mol Cell Cardiol*. 2007;43:636-647.

93. Watanabe H, Koopmann TT, Le Scouarnec S, Yang T, Ingram CR, Schott JJ, Demolombe S, Probst V, Anselme F, Escande D, Wiesfeld AC, Pfeufer A, Kaab S, Wichmann HE, Hasdemir C, Aizawa Y, Wilde AA, Roden DM and Bezzina CR. Sodium channel beta1 subunit mutations associated with Brugada syndrome and cardiac conduction disease in humans. *J Clin Invest*. 2008;118:2260-2268.

94. Ogawa R, Kishi R, Takagi A, Sakaue I, Takahashi H, Matsumoto N, Masuhara K, Nakazawa K, Kobayashi S, Miyake F and Echizen H. A novel microsatellite polymorphism of sodium channel beta1-subunit gene (SCN1B) may underlie abnormal cardiac excitation manifested by coved-type ST-elevation compatible with Brugada syndrome in Japanese. *Int J Clin Pharmacol Ther*. 2010;48:109-119.

95. Hu D, Barajas-Martinez H, Medeiros-Domingo A, Crotti L, Veltmann C, Schimpf R, Urrutia J, Alday A, Casis O, Pfeiffer R, Burashnikov E, Caceres G, Tester DJ, Wolpert C, Borggrefe M, Schwartz P, Ackerman MJ and Antzelevitch C. A novel rare variant in SCN1Bb linked to Brugada syndrome and SIDS by combined modulation of Na(v)1.5 and K(v)4.3 channel currents. *Heart Rhythm*. 2012;9:760-769.

96. Olesen MS, Holst AG, Svendsen JH, Haunso S and Tfelt-Hansen J. SCN1Bb R214Q found in 3 patients: 1 with Brugada syndrome and 2 with lone atrial fibrillation. *Heart Rhythm*. 2012;9:770-773.

97. Ricci MT, Menegon S, Vatrano S, Mandrile G, Cerrato N, Carvalho P, De Marchi M, Gaita F, Giustetto C and Giachino DF. SCN1B gene variants in Brugada Syndrome: a study of 145 SCN5A-negative patients. *Sci Rep*. 2014;4:6470.

98. Lin X, O'Malley H, Chen C, Auerbach D, Foster M, Shekhar A, Zhang M, Coetzee W, Jalife J, Fishman GI, Isom L and Delmar M. Scn1b deletion leads to increased tetrodotoxin-sensitive sodium current, altered intracellular calcium homeostasis and arrhythmias in murine hearts. *J Physiol*. 2015;593:1389-1407.

99. Aoki H, Nakamura Y, Ohno S, Makiyama T and Horie M. Cardiac conduction defects and Brugada syndrome: A family with overlap syndrome carrying a nonsense SCN5A mutation. *J Arrhythm*. 2017;33:35-39.

SCN2B

100. Watanabe H, Darbar D, Kaiser DW, Jiramongkolchai K, Chopra S, Donahue BS, Kannankeril PJ and Roden DM. Mutations in sodium channel beta1- and beta2-subunits associated with atrial fibrillation. *Circ Arrhythm Electrophysiol*. 2009;2:268-275.

101. Riuro H, Beltran-Alvarez P, Tarradas A, Selga E, Campuzano O, Verges M, Pagans S, Iglesias A, Brugada J, Brugada P, Vazquez FM, Perez GJ, Scornik FS and Brugada R. A missense mutation in the sodium channel beta2 subunit reveals SCN2B as a new candidate gene for Brugada syndrome. *Hum Mut*. 2013;34:961-966.

102. Bao Y, Willis BC, Frasier CR, Lopez-Santiago LF, Lin X, Ramos-Mondragon R, Auerbach DS, Chen C, Wang Z, Anumonwo J, Valdivia HH, Delmar M, Jalife J and Isom LL. Scn2b Deletion in Mice Results in Ventricular and Atrial Arrhythmias. *Circ Arrhythm Electrophysiol*. 2016;9:1-30.

SCN3B

103. Morgan K, Stevens EB, Shah B, Cox PJ, Dixon AK, Lee K, Pinnock RD, Hughes J, Richardson PJ, Mizuguchi K and Jackson AP. beta 3: an additional auxiliary subunit of the voltage-sensitive sodium channel that modulates channel gating with distinct kinetics. *Proc Natl Acad Sci*. 2000;97:2308-2313.

104. Fahmi AI, Patel M, Stevens EB, Fowden AL, John JE, 3rd, Lee K, Pinnock R, Morgan K, Jackson AP and Vandenberg JI. The sodium channel beta-subunit SCN3b modulates the kinetics of SCN5a and is expressed heterogeneously in sheep heart. *J Physiol*. 2001;537:693-700.

105. Maier SK, Westenbroek RE, McCormick KA, Curtis R, Scheuer T and Catterall WA. Distinct subcellular localization of different sodium channel alpha and beta subunits in single ventricular myocytes from mouse heart. *Circulation*. 2004;109:1421-1427.

106. Ko SH, Lenkowski PW, Lee HC, Mounsey JP and Patel MK. Modulation of Na(v)1.5 by beta1-- and beta3-subunit co-expression in mammalian cells. *Pflugers Arch*. 2005;449:403-412.

107. Hakim P, Gurung IS, Pedersen TH, Thresher R, Brice N, Lawrence J, Grace AA and Huang CL. Scn3b knockout mice exhibit abnormal ventricular electrophysiological properties. *Prog Biophys Mol Biol*. 2008;98:251-266.

108. Hu D, Barajas-Martinez H, Burashnikov E, Springer M, Wu Y, Varro A, Pfeiffer R, Koopmann TT, Cordeiro JM, Guerchicoff A, Pollevick GD and Antzelevitch C. A mutation in the beta 3 subunit of the cardiac sodium channel associated with Brugada ECG phenotype. *Circ Cardiovasc Genet*. 2009;2:270-278.

109. Hakim P, Brice N, Thresher R, Lawrence J, Zhang Y, Jackson AP, Grace AA and Huang CL. Scn3b knockout mice exhibit abnormal sino-atrial and cardiac conduction properties. *Acta Physiol*. 2010;198:47-59.

110. Olesen MS, Jespersen T, Nielsen JB, Liang B, Moller DV, Hedley P, Christiansen M, Varro A, Olesen SP, Haunso S, Schmitt N and Svendsen JH. Mutations in sodium channel beta-subunit SCN3B are associated with early-onset lone atrial fibrillation. *Cardiovasc Res*. 2011;89:786-793.

111. Ishikawa T, Takahashi N, Ohno S, Sakurada H, Nakamura K, On YK, Park JE, Makiyama T, Horie M, Arimura T, Makita N and Kimura A. Novel SCN3B mutation associated with brugada syndrome affects intracellular trafficking and function of Nav1.5. *Circ J*. 2013;77:959-967.

112. Okata S, Yuasa S, Suzuki T, Ito S, Makita N, Yoshida T, Li M, Kurokawa J, Seki T, Egashira T, Aizawa Y, Kodaira M, Motoda C, Yozu G, Shimojima M, Hayashiji N, Hashimoto H, Kuroda Y, Tanaka A, Murata M, Aiba T, Shimizu W, Horie M, Kamiya K, Furukawa T and Fukuda K. Embryonic type Na+ channel beta-subunit, SCN3B masks the disease phenotype of Brugada syndrome. *Sci Rep*. 2016;6:34198.

SCN5A

113. Chen Q, Kirsch GE, Zhang D, Brugada R, Brugada J, Brugada P, Potenza D, Moya A, Borggrefe M, Breithardt G, Ortiz-Lopez R, Wang Z, Antzelevitch C, O'Brien RE, Schulze-Bahr E, Keating MT, Towbin JA and Wang Q. Genetic basis and molecular mechanism for idiopathic ventricular fibrillation. *Nature*. 1998;392:293-296.

114. Bezzina C, Veldkamp MW, van Den Berg MP, Postma AV, Rook MB, Viersma JW, van Langen IM, Tan-Sindhunata G, Bink-Boelkens MT, van Der Hout AH, Mannens MM and Wilde AA. A single Na(+) channel mutation causing both long-QT and Brugada syndromes. *Circ Res*. 1999;85:1206-1213.

115. Rook MB, Bezzina Alshinawi C, Groenewegen WA, van Gelder IC, van Ginneken AC, Jongsma HJ, Mannens MM and Wilde AA. Human SCN5A gene mutations alter cardiac sodium channel kinetics and are associated with the Brugada syndrome. *Cardiovasc Res*. 1999;44:507-517.

116. Deschenes I, Baroudi G, Berthet M, Barde I, Chalvidan T, Denjoy I, Guicheney P and Chahine M. Electrophysiological characterization of SCN5A mutations causing long QT (E1784K) and Brugada (R1512W and R1432G) syndromes. *Cardiovasc Res*. 2000;46:55-65.

117. Vatta M, Dumaine R, Antzelevitch C, Brugada R, Li H, Bowles NE, Nademanee K, Brugada J, Brugada P and Towbin JA. Novel mutations in domain I of SCN5A cause Brugada syndrome. *Mol Genet Metab*. 2002;75:317-324.

118. Schulze-Bahr E, Eckardt L, Breithardt G, Seidl K, Wichter T, Wolpert C, Borggrefe M and Haverkamp W. Sodium channel gene (SCN5A) mutations in 44 index patients with Brugada syndrome: different incidences in familial and sporadic disease. *Hum Mut*. 2003;21:651-652.

119. Hong K, Guerchicoff A, Pollevick GD, Oliva A, Dumaine R, de Zutter M, Burashnikov E, Wu YS, Brugada J, Brugada P and Brugada R. Cryptic 5' splice site activation in SCN5A associated with Brugada syndrome. *J Mol Cell Cardiol*. 2005;38:555-560.

120. Kapplinger JD, Tester DJ, Alders M, Benito B, Berthet M, Brugada J, Brugada P, Fressart V, Guerchicoff A, Harris-Kerr C, Kamakura S, Kyndt F, Koopmann TT, Miyamoto Y, Pfeiffer R, Pollevick GD, Probst V, Zumhagen S, Vatta M, Towbin JA, Shimizu W, Schulze-Bahr E, Antzelevitch C, Salisbury BA, Guicheney P, Wilde AA, Brugada R, Schott JJ and Ackerman MJ. An international compendium of mutations in the SCN5A-encoded cardiac sodium channel in patients referred for Brugada syndrome genetic testing. *Heart Rhythm*. 2010;7:33-46.

SLMAP

121. Wigle JT, Demchyshyn L, Pratt MA, Staines WA, Salih M and Tuana BS. Molecular cloning, expression, and chromosomal assignment of sarcolemmal-associated proteins. A family of acidic amphipathic alpha-helical proteins associated with the membrane. *J Biol Chem*. 1997;272:32384-32394.

122. Guzzo RM, Salih M, Moore ED and Tuana BS. Molecular properties of cardiac tail-anchored membrane protein SLMAP are consistent with structural role in arrangement of excitation-contraction coupling apparatus. *Am J Physiol Heart Circ Physiol*. 2005;288:H1810-819.

123. Ishikawa T, Sato A, Marcou CA, Tester DJ, Ackerman MJ, Crotti L, Schwartz PJ, On YK, Park JE, Nakamura K, Hiraoka M, Nakazawa K, Sakurada H, Arimura T, Makita N and Kimura A. A novel disease gene for Brugada syndrome: sarcolemmal membrane-associated protein gene mutations impair intracellular trafficking of hNav1.5. *Circ Arrhythm Electrophysiol*. 2012;5:1098-1107.

124. Nader M, Westendorp B, Hawari O, Salih M, Stewart AF, Leenen FH and Tuana BS. Tail-anchored membrane protein SLMAP is a novel regulator of cardiac function at the sarcoplasmic reticulum. *Am J Physiol Heart Circ Physiol*. 2012;302:H1138-1145.

TRPM4

125. Stallmeyer B, Zumhagen S, Denjoy I, Duthoit G, Hebert JL, Ferrer X, Maugenre S, Schmitz W, Kirchhefer U, Schulze-Bahr E, Guicheney P and Schulze-Bahr E. Mutational spectrum in the Ca(2+)--activated cation channel gene TRPM4 in patients with cardiac conductance disturbances. *Hum Mut*. 2012;33:109-117.

126. Liu H, Chatel S, Simard C, Syam N, Salle L, Probst V, Morel J, Millat G, Lopez M, Abriel H, Schott JJ, Guinamard R and Bouvagnet P. Molecular genetics and functional anomalies in a series of 248 Brugada cases with 11 mutations in the TRPM4 channel. *PloS one*. 2013;8:e54131.

127. Demion M, Thireau J, Gueffier M, Finan A, Khoueiry Z, Cassan C, Serafini N, Aimond F, Granier M, Pasquie JL, Launay P and Richard S. Trpm4 gene invalidation leads to cardiac hypertrophy and electrophysiological alterations. *PloS one*. 2014;9:e115256.

128. Kruse M and Pongs O. TRPM4 channels in the cardiovascular system. *Curr Opin Pharmacol*. 2014;15:68-73.

129. Mathar I, Kecskes M, Van der Mieren G, Jacobs G, Camacho Londono JE, Uhl S, Flockerzi V, Voets T, Freichel M, Nilius B, Herijgers P and Vennekens R. Increased beta-adrenergic inotropy in ventricular myocardium from Trpm4-/- mice. *Circ Res*. 2014;114:283-294.

130. Guinamard R, Bouvagnet P, Hof T, Liu H, Simard C and Salle L. TRPM4 in cardiac electrical activity. *Cardiovasc Res*. 2015;108:21-30.

131. Gualandi F, Zaraket F, Malagu M, Parmeggiani G, Trabanelli C, Fini S, Dang X, Wei X, Fang M, Bertini M, Ferrari R and Ferlini A. Mutation Load of Multiple Ion Channel Gene Mutations in Brugada Syndrome. *Cardiol*. 2017;137:256-260.
